# Young Aboriginal people’s engagement with STI testing in the Northern Territory, Australia

**DOI:** 10.1186/s12889-020-08565-0

**Published:** 2020-04-06

**Authors:** Stephen Bell, Peter Aggleton, James Ward, Walbira Murray, Bronwyn Silver, Andrew Lockyer, Tellisa Ferguson, Christopher K. Fairley, David Whiley, Nathan Ryder, Basil Donovan, Rebecca Guy, John Kaldor, Lisa Maher

**Affiliations:** 1 Kirby Institute for infection and immunity in society, Level 6, Wallace Wurth Building, UNSW Sydney, Sydney, NSW 2052 Australia; 2 Centre for Social Research in Health, UNSW Sydney, Sydney, Australia; 3 College of Arts and Social Sciences, The Australian National University, Canberra, Australia; 4 School of Public Health, University of Queensland, Brisbane, Australia; 5 Central Australian Aboriginal Congress Aboriginal Corporation, Alice Springs, Australia; 6 Melbourne Sexual Health Centre, Melbourne, Australia; 7 Central Clinical School, Monash University, Melbourne, Australia; 8 University of Queensland, Brisbane, Australia; 9 Pacific Clinic Newcastle, Hunter New England Sexual Health, Newcastle, Australia; 10 School of Medicine and Public Health, The University of Newcastle, Newcastle, Australia; 11 Burnet Institute, Melbourne, Australia

**Keywords:** STI testing, Young people, Aboriginal, Indigenous, Qualitative, Australia

## Abstract

**Background:**

Australian surveillance data document higher rates of sexually transmissible infections (STIs) among young Aboriginal people (15**–**29 years) in remote settings than non-Aboriginal young people. Epidemiological data indicate a substantial number of young Aboriginal people do not test for STIs. Rigorous qualitative research can enhance understanding of these findings. This paper documents socio-ecological factors influencing young Aboriginal people’s engagement with clinic-based STI testing in two remote settings in the Northern Territory, Australia.

**Methods:**

In-depth interviews with 35 young Aboriginal men and women aged 16–21 years; thematic analysis examining their perceptions and personal experiences of access to clinic-based STI testing.

**Results:**

Findings reveal individual, social and health service level influences on willingness to undertake clinic-based STI testing. Individual level barriers included limited knowledge about asymptomatic STIs, attitudinal barriers against testing for symptomatic STIs, and lack of skills to communicate about STIs with health service staff. Social influences both promoted and inhibited STI testing. In setting 1, local social networks enabled intergenerational learning about sexual health and facilitated accompanied visits to health clinics for young women. In setting 2, however, social connectedness inhibited access to STI testing services. Being seen at clinics was perceived to lead to stigmatisation among peers and fear of reputational damage due to STI-related rumours. Modalities of health service provision both enhanced and inhibited STI testing. In setting 1, outreach strategies by male health workers provided young Aboriginal men with opportunities to learn about sexual health, initiate trusting relationships with clinic staff, and gain access to clinics. In setting 2, barriers were created by the location and visibility of the clinic, appointment procedures, waiting rooms and waiting times. Where inhibitive factors at the individual, social and health service levels exist, young Aboriginal people reported more limited access to STI testing.

**Conclusions:**

This is the first socio-ecological analysis of factors influencing young Aboriginal people’s willingness to undertake testing for STIs within clinics in Australia. Strategies to improve uptake of STI testing must tackle the overlapping social and health service factors that discourage young people from seeking sexual health support. Much can be learned from young people’s lived sexual health experiences and family- and community-based health promotion practices.

## Background

Young Aboriginal and Torres Strait Islander (‘Aboriginal’ from here on) people resident in regional and remote centres in Australia are more likely than their non-Aboriginal age counterparts to acquire sexually transmissible infections (STIs) [1]. In 2017, surveillance data indicates higher notification rates for Aboriginal populations than non-Indigenous populations for Chlamydia (1194 vs. 427 per 100,000 people), gonorrhoea (627 vs. 96 per 100,000 people) and infectious syphilis (102.5 vs 15.5 per 100,000 people). STI notification rates are up to 5, 30 and 50 times higher respectively for Chlamydia, gonorrhoea and infectious syphilis respectively in remote and very remote areas, and were higher among Indigenous young people aged 15–29 years compared to older Indigenous people and non-Indigenous age peers [1]. In hyperendemic areas such as the Northern Territory, Australia – where the STI prevalence and incidence rates among young Aboriginal people are persistently high [2–5] despite the existence of primary health care, sexual health services and programs, and policy that prioritises STI control [6–8] – improvement is dependent on high population coverage of regular repeat STI testing and earlier detection of infection and follow up.

Given high STI prevalence and incidence rates in the Northern Territory, national guidelines recommend twice annual testing, timely treatment and regular re-testing for STIs for sexually active young Aboriginal people aged 15–35 years [6, 9]. In this setting most STI testing occurs through opportunistic screening, which relies on strategies that improve health seeking behaviours and the provision of safe, trustworthy clinic environments for clients. However, behavioural surveys and epidemiological research illustrates that STI testing and retesting after STI treatment is more common among young Aboriginal women than young men [2, 3, 8, 10], and that younger people aged 16–19 years who have the highest STI prevalence are less likely to test than those aged 20–29 years [10]. There is urgent need to understand how earlier, more frequent engagement in regular STI testing can be achieved among young Aboriginal men and women [8, 11–13].

Rigorous qualitative research can provide in-depth insight into the factors underlying findings identified in survey-based and epidemiological research [14]. Yet there exists a paucity of qualitative research examining sexual health among young Aboriginal Australians [15]. No published qualitative literature explicitly documents factors influencing young Aboriginal people’s engagement with clinic-based STI testing, from their own perspectives. Barriers to STI testing are documented only briefly in papers focusing more generally on youth relationships and sexual health literacy [16], sexual risk and resilience practices [17–19], perceptions of health [20], capacity building of sexual health workers [21], engaging young people in sexual health research [20], and sexual health education [22]. This literature highlights young Aboriginal people’s concerns about a lack of confidentiality in health service provision; feelings of shyness, embarrassment and shame; the potential for reputational damage associated with STIs; and anxiety about waiting times and interactions with clinic staff [16, 17, 19–22]. A more comprehensive understanding of these issues increases opportunities to provide young Aboriginal women and men with access to culturally safe sexual health support – where culturally safety relates to environments that are free from assault, challenge or denial of identity and needs, in which services are provided on the basis of shared respect, meaning, knowledge and experience, and where all people are listened to and treated with dignity [23] – as advocated for by the ‘Fifth National Aboriginal and Torres Strait Islander Australian Blood-borne Viruses and Sexually Transmissible Infections Strategy 2018-2022’ [24].

Drawing on data collected in 2015–16, this is the first qualitative study examining young Aboriginal people’s perceptions of the broad socio-ecological [25] factors influencing their uptake of clinic-based STI testing in two remote settings in the Northern Territory, Australia. The aim of this paper is to provide case study evidence from two remote settings about the social processes influencing uptake of STI testing among male and female Aboriginal young people aged 16–21 years.

## Methods

This interpretive qualitative study was a component of a larger study evaluating strategies to increase STI testing among Aboriginal people aged 16–29 years in the Northern Territory, Australia. Between October 2015 and November 2016, 35 young Aboriginal people – women and men, aged 16–21 years, resident in two remote settings^1^ in the Northern Territory – participated in individual in-depth interviews. Findings from the study were shared in full with participating health services and informed the development of two strategies aimed at increasing clinic-based STI testing among young Aboriginal people, one of which is currently being evaluated. We used the COREQ checklist to report the methods we used in this study (Additional file 1: COREQ checklist).

Given that people in this age group are at highest risk of STIs and the hardest to engage in clinic-based STI testing [1, 10], and the existence of clear gender differences in accessing STI testing services [2, 3, 8, 10], young Aboriginal people were sampled purposively [26] to reflect diversity by age and gender in each setting. Young people were eligible to participate if they identified as being Aboriginal, were aged 16–21 years and resident in each setting. Recruitment occurred face to face via introductions arranged by representatives of local health services and youth development organisations in setting 1, and by youth researchers in setting 2.

Semi-structured in-depth interviews were conducted with young people, in person on an individual basis. In setting 1, interviews were conducted by two experienced adult researchers – SB (PhD, social science, non-Indigenous male researcher with no prior connection to either research setting) and WM (Research Officer, Aboriginal woman with previous youth work experience in this location). In this setting, respondents were not known to the interviewers prior to data collection. In setting 2, informed by an established peer research study design [27], interviews were conducted by two adult researchers – SB and AL (Research Officer, Aboriginal man with previous youth work experience in this location) and six project-specific employed Aboriginal youth researchers aged 16–19 years who were recruited to undertake research with other young people in their local social networks. They participated in a four-day qualitative research training workshop covering semi-structured in-depth interviewing; applied research ethics, with a particular emphasis on confidentiality, anonymity, informed voluntary consent and the use of participant information sheets and consent forms; the use of a digital recorder; data management; and interviewee recruitment. They also received a further 14 days of intensive research support from SB and AL to enhance interviewing skills as data collection progressed. Youth researchers were known to AL but not known to SB prior to data collection; interviewees were known to youth researchers prior to data collection.

The topic guide (Additional file 2: Topic guide) was piloted with and adapted by youth researchers in setting 2 and covered three themes, including young people’s sexual experiences and relationships; STIs and risk practices; prevention, risk reduction and STI testing and treatment. The guide was designed around the use of ‘third person interviewing’ – i.e. not asking the interviewee to talk directly about themselves but about ‘other people like themselves’. This served several purposes in the two research settings: first, it facilitated more comfortable, less intimidating conversations about sexual health, a topic which many participants had rarely spoken openly about with other people beyond trusted networks; second, it enabled the collection of data about diverse perspectives and experiences that young people can experience in these settings; and third, it elicited the local meanings and consequences that young people attribute to and associate with social and sexual practices in these settings [27]. Despite using a third person interviewing approach, all young participants also spoke about personal experiences, or the lived experiences of friends and peers. As such, in this paper we report on both young participants’ perceptions and experiences of engaging with STI testing services in these settings. Interviews took place in audio-private settings (e.g. private rooms in youth and sports organisations; at home on verandas and in gardens; under trees in public spaces), lasted between 25 and 90 min and were conducted in English. Debriefing occurred after each interview.

All interviews were audio-recorded, transcribed verbatim, de-identified, checked for accuracy, and imported into QSR NVIVO V.12 qualitative data analysis software. Transcripts were not returned to participants for comment or correction. However, prior to analysis, and with support from SB, interview audio files were reviewed by adult and youth Aboriginal researchers involved in data collection for initial interpretation of data. Thereafter a thematic analysis was led by the first author, with support from JW, PA and LM, using a system of open and axial coding [28] to examine young people’s perceptions of access to health services for STI testing. The analysis used a socio-ecological framework [25] to examine individual (e.g. knowledge, attitudes), social (e.g. informal interactions within intimate, peer and family relationships), and health service (e.g. formal systems of health service delivery, clinic location) factors influencing access to STI testing. Further inductive analysis was undertaken to examine differences within these themes.

Participants were remunerated with either a AUD$30 local store voucher or AUD$30 mobile phone credit. Ethical approval was received from the Central Australian Human Research Ethics Committee (HREC 15–314).

## Results

Thirty-five young people participated in this study. Fifteen participants (6 women, 9 men) aged 16–21 years were resident in setting 1; 20 participants (8 women, 12 men) aged 16–19 years were resident in setting 2. During interviews, one third of participants (3 young woman and 8 young men) indicated personal experience of STI testing. Drawing only on young people’s perspectives and experiences, we outline socio-ecological influences at individual, societal and health service levels that can encourage and inhibit clinic-based STI testing.

### Individual influences

A lack of knowledge about STIs and the need for regular STI testing was identified by young men in setting 1 and by young women and men in setting 2.

Young Aboriginal people are not really aware of STIs and they don’t know whether, if they have them or not.

(Interviewee 13, young woman, 16–18 years, setting 2)

An absence of symptoms led some participants to believe they were not in need of STI testing. Young women pointed to the need for education to raise understanding about asymptomatic STIs.

I don’t even think they know that they have to have regular check-ups. It’s lack of knowledge, lack of information. There’s not even any brochures or pamphlets on those kind of things.

(Interviewee 17, young woman, 16–18 years, setting 2)

Young people in both settings described a range of symptoms – both perceived and personally experienced – relating to STIs. Young women referred to ‘feeling bad in my tummy’, ‘sore lips’, ‘feel sick’, having [a] rash’, ‘itchiness’ and ‘feeling really sick in private parts’. Young men described more specific symptoms such as ‘boils’, ‘pussy penis’ and having ‘gold stuff’ and ‘green stuff’.

Even with symptomatic STIs, young people in the town indicated that access to health services was often inhibited or delayed. Young men described attitudinal barriers that contribute to a passive approach to seeking treatment. One participant stated, “sometimes we just can’t be bothered” (Interviewee 1, young man, 16–18 years, setting 2), while another said “some [young men] might not even care” (Interviewee 10, young man, 16–18 years, setting 2). Young women reported hoping that symptoms would disappear.

Most of them would just let it ride and hope that it will clear up in a few days or so… Most of them just try to shrug it off and just hope it goes away.

(Interviewee 17, young woman, 16–18 years, setting 2)

In contrast to setting 2, young people in setting 1 reported a greater likelihood of seeking treatment at their local clinic when symptoms were present and persistent.

*How does someone know if they have a sexual sickness?* They might found out at the clinic. If that young girl had sickness they might find out at the clinic. They go to treat sickness.

(Interviewee 30, young woman, 19–21 years, setting 1)

*If a young person gets sexual sickness, what do they do?* They’ll get pain. They think about clinic when they get infection. *And the clinic always helps?* Yeah *Have you, have you used the clinic here?* Yeah I been go to clinic here. For check up. I go self. *Because you had pain?* Yeah, it been get painful.

(Interviewee 23, young man, 19–21 years, setting 1)

### Social influences

Participants in setting 2 worried about encountering friends or family at the clinic and being asked questions about why they are there. They described their concerns of embarrassment and shame about not being able to keep a clinic visit a secret and concern about how to respond.

*Might people go and get help, or go and get treatment?* If they’re game enough, but I doubt it because they’ll be scared that they’ll get caught out. Unless they talk to their parents about it first. *How might they get caught out?* Might get seen going to the clinic and then asked question after question after question until it slips out and you tell them by accident. And if there’s nothing wrong with you, it just looks really suspicious.

(Interviewee 7, young man, 16–18 years, setting 2)

Young people also described the tendency for people to assume that if a young person is seen in a clinic setting it must be for sexual health problems; a potential indication of sexual activity that may not be socially acceptable.

People in [setting 2] naturally assume that something’s wrong with them, straight away. Like, ‘oh, they’re at the clinic, they must be sick’, ‘oh, oh, look they’re getting her bits checked’, ‘oh, look, they’re getting his bits checked’, ‘he’s probably got a disease’, ‘she’s probably got a disease, yuk’.

(Interviewee 17, young woman, 16–18 years, setting 2)

In setting 2, young people described the possibility that being seen at a clinic might lead to teasing and name calling. Other possible consequences included gossip and reputational damage as rumours spread to wider community members and parents, triggering parental disappointment, punishment at home, social exclusion from peer and friendship groups, and emotional turmoil for the victims of gossip.

*Do young people know about STIs and the services that are around that can help them with that sort of stuff?* Um, yeah. They know it, but I reckon they would think… it’s not much privacy so they’re too shame to go see them. *Where are you talking about?* [Aboriginal health service] men’s clinic, round here. *Who might see them?* Family, mostly family… If one person sees them, they’re like “oh I seen him going into that place over there. He’s probably got disease”. And start talking shit like that.

(Interviewee 10, young man, 16–18 years, setting 2)

These negative social responses inhibited health service use but were described as a normal, expected part of life in this particular location. As one respondent put it, ‘This is [name of place]. Everybody talks and talks’ (Interviewee 16, young woman, 19–21 years, setting 2).

Young people in setting 1 also acknowledged feelings of shame and embarrassment associated with contracting STIs. However, in contrast to young people in setting 2, young women in setting 1 described greater social support for their use of the local health service. Women’s nights were organised by adult women and local youth services to enable younger and older women to talk collectively about health issues such as menstruation, pregnancy and pregnancy prevention. Use of the local clinic for STI testing could be passed off as related to these broader women’s health concerns. Young women in this setting also described how sisters, cousins, aunts, mothers, grandmothers and close friends accompanied them to clinic visits.

For us mob, like, for [women], we get all the [women] to come down ‘n’ sit down ‘n’ talk story about women’s you know? But I don’t think that’s happening for the boys. *If one of your friends thought they might have a sickness, what would you say to them or tell them to do?* Ah I would just help them, take her down to the clinic. I’ll help her. If she woulda had that sickness for a long time, she woulda come up to me and tell me, ‘I’m really scared but I want you to help me, to take me down clinic’. ‘cause I know she wouldn’t go herself to the clinic and she’ll feel really shame.

(Interviewee 35, young woman, 16–18 years, setting 1)

Young Aboriginal men in setting 1 did not report the same range of social support as young women. They tended to keep any STI symptoms private but, in contrast to young men in setting 2, showed greater willingness to engage with STI testing and treatment for symptomatic STIs at the local health service as they could do so more discreetly.

*A young fella say 16, 17, 19 if he gets infection, who does he talk to? To get some help?* Um… I dunno *So if, if you got, if you got an infection, who would you talk to about it?* Um, I’d go to the clinic straight away. *Ah, you tell your friends?* No. Bit secret*. Keep it secret?* Yeah*. Why do you want to keep it secret?* ‘Cause some people… not telling… just better that way. They keeping secret, and own way going to clinic.

(Interviewee 23, young man, 19–21 years, setting 1)

### Health service influences

Influences that inhibited STI testing were associated with systems of health service provision. Two young men in setting 1 noted the lack of a separate entrance and waiting room for men and women. Young women in both settings described how the design of clinic waiting rooms facilitated being seen (and attendant gossip), particularly in setting 2 where waiting times are longer.

*What about testing in the main clinic at [clinic]?* Too many big eyes. *Too many big eyes?* Big eyes, big ears. Yeah. It’s too small. You need to be somewhere where no one is focusing on you. Where they’re focusing on everybody else as well.

(Interviewee 16, young woman, 19–21 years, setting 2)

Participants in setting 2 expressed concern about the location of the STI testing services, on a busy main road. The inability to pre-book an appointment here also increased the chance of being seen near, entering or in these clinics.

On the other hand, the design of health service provision could enhance STI testing. Young men in setting 1 reported positive interaction with male health workers in group meetings where they learned about STIs and other men’s health issues. One young man (Interviewee 22, 16–18 years, setting 1) described how he had first learned about condoms from the remote men’s health outreach team during a talk at a barbecue after football training. Another described health workers taking “all the fellas to the [local clinic]”, “putting up posters” and talking openly about sexual health.

You’ve met [remote men’s health outreach worker’s name ] before? *Ah I know him from [town].* ‘Cause he used to come every day to clinic and he used to show all the healthy stuff to the fellas and he used to pick up all the fellas and take ‘em to the clinic. *So do you remember the things that he taught you?* Yeah like healthy, like if you like, if you have sex with all the girls you got to go to clinic to have a check hey. And you gotta use like, safety things [condoms]. He used to talk like healthy things at hospital. And like this thing, like after healthy stories and after that we used to eat them sausage at BBQ. *There were lot, lots of young fellas that, that went?* Yeah lots of young fellas and big fellas and old mans. All together. We used to always to go to the hospital and he used to talk like healthy stuff.

(Interviewee 27, young man, 16–18 years, setting 1)

In this setting, health outreach workers were able to establish a context in which young men were willing to seek STI testing and support at the local health service – for routine testing, a check-up after sex without a condom, or symptomatic STIs – as the place was safe, the procedures known, and the people trusted.

However, young people in setting 2 described very different experiences. For them, there was significant concern about interacting with health workers about sexual health issues because they did not know how to communicate about STIs and feared having a conversation with someone they have never talked to before.

Sometimes young people do get shame. ‘Cos it’s just how they are. It’s how they are. They’ve never talked to anyone about it before.

(Interviewee 18, young woman, 16–18 years, setting 2)

They mainly try to avoid [using STI testing services]. Because it’s shame job. Walking in and telling someone you don’t even know… the shame of telling the doctor, cos you don’t know them, and you don’t know what they’re like. You’re just going to walk in and tell some random person what’s going on?!

(Interviewee 7, young man, 16–18 years, setting 2)

These quotes illustrate how the confluence of individual, social and health service influences can inhibit the uptake of STI testing and treatment – individual influences associated with lack of knowledge and communication skills; social influences associated with a lack of support or encouragement from friends and family, and confidentiality concerns; and institutional influences due to a lack of opportunity to build trust in health workers or familiarity of the clinic space.

## Discussion

This is the first qualitative study to examine – from the perspectives of young Aboriginal Australians’ resident in remote Australia, and focussing on gender differences – multiple factors influencing young Aboriginal people’s engagement with clinic-based STI testing. We do so using socio-ecological analysis to highlight the individual, social and health service influences in two contrasting remote settings in the Northern Territory. Our findings illustrate the importance of strong family and community support for health service use, youth-friendly design of health service spaces and appointment procedures, efforts to minimise sexual stigma, and innovative sexual health outreach programs, particularly for young Aboriginal men, to reduce high rates of STIs and enhance engagement in clinic-based STI testing.

At an individual level, young women and men in both settings identified barriers to STI testing linked to limited knowledge and awareness of the transmission and prevention of STIs, and their often asymptomatic nature. Similar findings are reported in studies with other young people in Australia [16–18, 22, 29–31]. Some participants identified further attitudinal barriers that inhibited or delayed STI testing for symptomatic STIs and a lack of skills to communicate about STIs with health service workers. Among other things, these findings reaffirm the inadequacy of skills-based relationships and sexuality education for young Aboriginal people in diverse settings [15, 17, 22, 24, 32, 33], which is identified as an urgent priority in the latest national strategy [24].

Beyond the individual level, social connectedness and interaction – especially with friends, peers and family – had divergent effects. In the remote community, women’s nights provided a safe space for young women to interact with older women and share advice about women’s health issues. Such social interactions outside clinic spaces enhanced young women’s individual knowledge and awareness of the issues. However, these informal social support networks also extended into clinic spaces. Accompanied visits to the clinic for women’s health issues – with support from sisters, cousins, aunts, mothers, grandmothers and close friends – enabled young women to gain experience of STI testing procedures under the guise of something else. In this setting, young women’s strong intergenerational, peer and family support networks reduced barriers associated with individual influences, limited the occurrence of social stigma associated with STIs, and enhanced access to and experience within clinic settings.

In contrast, for young people in setting 2, social connectedness and interaction within and outside clinic settings proved to be a barrier to STI testing. Without inter- and intragenerational social support, fear of being seen by, or having to converse with, peers, family or other community members in a clinic waiting room created anxiety and fear of being judged and maligned by peers and family. As reported in other studies in remote and regional Australia [22, 34], teasing, name-calling and gossip within peer networks, and the reputational damage associated with rumours that spread beyond to families were detrimental to STI testing among young people in this setting. Here, the social connections within health service settings led to social processes beyond the clinic which discouraged engagement in clinic-based STI testing.

Health systems delivery influenced young people’s engagement with STI testing. Health promotion outreach strategies – delivered via a partnership between the government clinic and the government-led remote men’s health team – were reported by young men in setting 1 to enhance their willingness to attend the local clinic for STI testing. Ongoing discussion with male health workers during social and sports activities outside the clinic setting, and accompanied visits through the clinic, enhanced social connectedness between young Aboriginal men and male health workers. These strategies brought health services and social influences together and provided opportunities for young men to learn about sexual health issues; experience how to communicate with health professionals; initiate trusting relationships with clinic staff; and become more comfortable accessing formal clinic spaces.

In contrast, young people in setting 2 reported fewer opportunities (outside of a formal, anxiety-provoking clinic visit) to interact with health workers or knowledgeable and supportive friends and family members. As a result, they were not able to acquire the knowledge, language and skills required to negotiate access to a busy, centrally located service in which privacy could not be assured, and lacked both confidence and trust to do so. Nor were they able to acquire the skills to communicate comfortably with other people about issues pertaining to STIs, sexual health and personal sexual practices, for fear of stigmatisation by others. Additionally, in setting 2, the geographical location of the clinic, the nature of the clinic space (i.e. entry points, waiting rooms), appointment procedures (i.e. pre-booked versus drop-in procedures) and waiting times prior to appointments enabled peer and family surveillance of young people’s clinic attendance.^2^ Young Aboriginal people’s fear of lack of confidentiality in health service provision, and concerns about waiting times and interactions with clinic staff have also been documented in other studies [19–21, 35]. Here, the exacerbation of social barriers within clinic spaces illustrates the complex challenges of providing private and confidential, but culturally and socially safe clinic-based STI testing.

### Limitations

While our sample of young people was large enough to allow ‘thematic data saturation’ [28], data collection was limited to a small sample of young people resident in two settings in the Northern Territory. Therefore, care must be taken to not generalise beyond these settings. In addition, information was not captured regarding the reasons why participants declined to participate. Data collection by a range of interviewers may have increased the variation among individual responses, although ‘internal reliability’ [36] was enhanced by interviewers working together during data interpretation and analysis processes to ensure rigour and consistency in data interpretation. Despite these limitations, study findings provide timely insights into the lived experiences of young Aboriginal people and their engagement with STI testing, signalling the range of socio-ecological factors that must be engaged with and addressed if testing is to be a success.

### Implications for programmes and services

Given the high prevalence, incidence and poor health outcomes associated with STIs, and the need for extremely high levels of STI testing and treatment in these settings, our findings – when used in collaboration with other available published evidence documenting the expertise of other stakeholders including family members, health workers and policy makers – have implications for strategies designed to enhance young people’s access to STI testing in remote settings. These will help meet priorities identified in the national strategy [24].

To overcome individual level barriers, action is needed to enhance young Aboriginal people’s communication skills, health seeking practices and understanding of asymptomatic STIs. Culturally responsive comprehensive relationships and sexuality education – in school, clinical, community and peer-based settings [24], and with a focus on health literacy, interpersonal and negotiation skills to navigate healthy relationships and access health services [37, 38] – would be beneficial, to upscale the limited current provision throughout the Northern Territory.

Action is also required to strengthen social support networks that include peers, families and other community members, and systems of health service provision to enhance young people’s access to and use of health services in remote settings. The women’s nights and the men’s health BBQs illustrate the value of activities that: (i) bring young people together in safe environments to talk about sexual health issues; (ii) involve supportive, trusted and influential family and community members in youth sexual health promotion activities; (iii) ‘nest’ STI-related services within broader systems of support for women’s and men’s health; (iv) enable young people to gain experience communicating with health workers in informal settings beyond the clinic environment. Accompanied clinic visits – with either health workers, friends, or trusted family members – can also facilitate culturally safe STI testing experiences.

There remain challenges to be faced however. These include how to reduce the social risks associated with young people’s clinic attendance, which is essential to the prevention, testing and management of STIs in this population [24]. Several approaches are worthy of further exploration in this regard including: (i) health worker community outreach work and accompanied visits to health services; (ii) the provision of youth-only sessions to limit youth interactions with adult family and community members within the clinic waiting room, and reduce individual risk by facilitating group-based testing; (iii) service re-design research to understand how to eliminate the risky moments of interaction between young and other clients in clinic settings; (iv) messaging about the harms associated with rumour, gossip and reputational damage in communication campaigns.

## Conclusions

This qualitative study highlights the complex socio-ecological influences affecting the engagement of young Aboriginal women and men in clinic-based STI testing in two remote settings in the Northern Territory. While individual level barriers are evident, strategies to improve engagement with STI testing must also tackle the social and health service factors that discourage young people from seeking sexual health support. Much can be learned from young people’s lived sexual health experiences in diverse settings and local Aboriginal forms of family- and community-based health promotion practice in communicating sexual health messages and facilitating supported access to health services.

## Supplementary information

**Additional file 1.** Consolidated criteria for reporting qualitative studies (COREQ): 32-item checklist.

**Additional file 2.** Discussion guide.

## Data Availability

The datasets generated and analysed during the current study are not publicly available as the research team did not seek permission from participants to share the data with third parties.

## Abbreviation

STIs: Sexually transmitted infections

## Footnotes

1 Deliberate strategies have been taken to protect the anonymity of the young Aboriginal people and health services involved in this study. Referred to only as settings 1 and 2 in this paper, neither location is named and limited social, cultural and demographic descriptions of each setting is provided.

2 Since 2016, major efforts have been made to decentralise health service provision in this setting. New satellite clinics have been opened in residential areas, which are designed to enhance confidentiality of clinic spaces, increase the ratio between staff and clients, and reduce waiting times.
