# Supplementary material for: Young Aboriginal people’s engagement with STI testing in the Northern Territory, Australia
Source: BMC Public Health. 2020 Apr 6;20:459. doi: 10.1186/s12889-020-08565-0 (PMC7137447; doi:10.1186/s12889-020-08565-0)
Supplement: Supplementary file 1 — Additional file 1. Consolidated criteria for reporting qualitative studies (COREQ): 32-item checklist. [file 12889_2020_8565_MOESM1_ESM.docx]

**Additional file 1: Consolidated criteria for reporting qualitative studies (COREQ): 32-item checklist**

Developed from:

Tong A, Sainsbury P, Craig J. Consolidated criteria for reporting qualitative research (COREQ): a 32-item checklist for interviews and focus groups. *International Journal for Quality in Health Care*. 2007. Volume 19, Number 6: pp. 349 – 357

| **No. Item** | **Guide questions/description** | **Reported on Page #** |
| --- | --- | --- |
| **Domain 1: Research team and reﬂexivity** | | |
| *Personal Characteristics* | | |
| 1. Inter viewer/facilitator | Which author/s conducted the interview or focus group? | Methods; page 5, lines 111-119. |
| 2. Credentials | What were the researcher’s credentials? | Methods; page 5, lines 112-117. |
| 3. Occupation | What was their occupation at the time of the study? | Methods; page 5, lines 112-117. |
| 4. Gender | Was the researcher male or female? | Methods; page 5, lines 111-117. |
| 5. Experience and training | What experience or training did the researcher have? | Methods; page 5-6, lines 111-126. |
| *Relationship with participants* | | |
| 6. Relationship established | Was a relationship established prior to study commencement? | Methods; page 5, lines 114-115 and lines 125-126. |
| 7. Participant knowledge of the interviewer | What did the participants know about the researcher? | Methods; page 5, lines 114-115 and lines 125-126. |
| 8. Interviewer characteristics | What characteristics were reported about the inter viewer/facilitator? | Methods; page 5, lines 112-126. |
| **Domain 2: study design** | | |
| *Theoretical framework* | | |
| 9. Methodological orientation and Theory | What methodological orientation was stated to underpin the study | Methods; page 4, lines 92-94 |
| *Participant selection* | | |
| 10. Sampling | How were participants selected? | Methods; page 5, lines 102-108. |
| 11. Method of approach | How were participants approached? e.g. face-to-face, telephone, mail, email | Methods; page 5, lines 102-108. |
| 12. Sample size | How many participants were in the study? | Methods; page 4-5, lines 94-97. |
| 13. Non-participation | How many people refused to participate or dropped out? Reasons? | Information not collected - this has been listed as a limitation |
| *Setting* | | |
| 14. Setting of data collection | Where was the data collected? e.g. home, clinic, workplace | Methods; page 6, paragraph 142-145. |
| 15. Presence of non-participants | Was anyone else present besides the participants and researchers? | Methods; page 5, lines 110-111. |
| 16. Description of sample | What are the important characteristics of the sample? e.g. demographic data, date | Methods; page 4-5, lines 94-96. |
| *Data collection* | | |
| 17. Interview guide | Were questions, prompts, guides provided by the authors? Was it pilot tested? | Methods; page 6, lines 128-145. |
| 18. Repeat interviews | Were repeat interviews carried out? If yes, how many? | Repeat interviews were not carried out. |
| 19. Audio/visual recording | Did the research use audio or visual recording to collect the data? | Methods; page 6, line 149. |
| 20. Field notes | Were ﬁeld notes made during and/or after the inter view or focus group? | Methods; page 6, lines 144-145. |
| 21. Duration | What was the duration of the inter views or focus group? | Methods; page 6, line 144. |
| 22. Data saturation | Was data saturation discussed? | Limitations; page 16, line 433 |
| 23. Transcripts returned | Were transcripts returned to participants for comment and/or correction? | Methods; page 6, lines 150-153 |
| **Domain 3: analysis and ﬁndings** | | |
| *Data analysis* | | |
| 24. Number of data coders | How many data coders coded the data? | Methods; page 6, line 154 |
| 25. Description of the coding tree | Did authors provide a description of the coding tree? | N/A |
| 26. Derivation of themes | Were themes identiﬁed in advance or derived from the data? | Methods; page 6-7, lines 154-160. |
| 27. Software | What software, if applicable, was used to manage the data? | Methods; page 6, line 150. |
| 28. Participant checking | Did participants provide feedback on the ﬁndings? | Methods; page 6, lines 150-153 |
| *Reporting* | | |
| 29. Quotations presented | Were participant quotations presented to illustrate the themes/ﬁndings? Was each quotation identiﬁed? e.g. participant number | Results |
| 30. Data and ﬁndings consistent | Was there consistency between the data presented and the ﬁndings? | Yes |
| 31. Clarity of major themes | Were major themes clearly presented in the ﬁndings? | Themes are outlined in the Discussion |
| 32. Clarity of minor themes | Is there a description of diverse cases or discussion of minor themes? | Yes included in Results and Discussion |
