# Supplementary material for: Young Aboriginal people’s engagement with STI testing in the Northern Territory, Australia
Source: BMC Public Health. 2020 Apr 6;20:459. doi: 10.1186/s12889-020-08565-0 (PMC7137447; doi:10.1186/s12889-020-08565-0)
Supplement: Supplementary file 2 — Additional file 2. Discussion guide. [file 12889_2020_8565_MOESM2_ESM.docx]

**Additional file 2: Discussion guide**

**INTERVIEW AIMS**

- Gather insight into Aboriginal young people’s knowledge, attitudes, practices and behaviours relating to sexual health, and their experiences of accessing and using local sexual health services;
- Identify the local language and terminology used by Aboriginal young people to frame sexual health, STIs and sexual health services.
- Initiate conversations about sex, talk positively about Aboriginal young people’s sexual experiences to help understand why Aboriginal young people have sex, get involved in sexual relationships, their sexual health support networks, any social expectations around Aboriginal young people’s sexual health, and any problems that might arise from sexual experiences.
- Find out what Aboriginal young people know about STIs, explore perceptions of risk, and examine what makes some Aboriginal young people vulnerable to poor sexual health experiences.
- Understand what Aboriginal young people do to prevent/reduce risk of sexual health problems and monitor sexual health, and examine young Aboriginal people’s use of STI testing and treatment services.

## INTERVIEW STRUCTURE (60 minute interview):

| **SECTION** | **TIMING** |
| --- | --- |
| **Introductions and ethics**  Researcher to introduce her/himself, the project, the nature of the interview and ethical issues, gain informed consent, and ask a couple of warm up questions | 10 mins |
| **Young people’s sexual experiences and relationships (theme 1)**  Initiate conversations about sex, talk positively about Aboriginal young people’s sexual experiences to help understand why Aboriginal young people have sex, get involved in sexual relationships, their sexual health support networks, any social expectations around Aboriginal young people’s sexual health, and any problems that might arise from sexual experiences. | 15 mins |
| **STIs and risk practices (theme 2)**  Find out what Aboriginal young people know about STIs, explore perceptions of risk, and examine what makes some Aboriginal young people vulnerable to poor sexual health experiences. | 15 mins |
| **Prevention, risk reduction and STI testing/treatment (theme 3)**  Understand what Aboriginal young people do to prevent/reduce risk of sexual health problems and monitor sexual health, and examine young Aboriginal people’s use of STI testing and treatment services. | 15 mins |
| **Conclusions**  Wrap up the interview | 5 mins |

## DETAILED DISCUSSION GUIDE

| **SECTION** | **TIMING** |
| --- | --- |
| **Introductions and ethics**  Greeting and warm welcome - Thank interviewee for agreeing to take part – mention that the discussion should last up to 60 minutes.  Introduce self  Run through PIS and CFs:  Explain for the safety of the research process there are some important issues that we need to introduce - focus on the participation information sheet and consent form, and explain that this is the process which we have to go through for all interviews conducted.  Explain that the aim of the research is to gather local expert views on how best to improve sexual health amongst young people locally, and that we will be speaking with a range of adult key informants as well as young people locally. Brief explanation of the study and the need for an interview with you and other young people.  Explain we are not asking about you, but about other people like you. We don't want information about your own lives. And we don’t use any names  Reassure interviewee of confidentiality and anonymity – we will not attribute comments to them through use of pseudonyms, and this means that there are no risks resulting from their involvement. No one will know you have taken part.  What will be done with the information  Stress there are no right or wrong answers – we are just interested in finding out her/his views and opinions. Explain that we have a series of open answer questions, and that the discussion will be directed by these and the responses provided.  Voluntary participation (no one has to take part) - You can stop the interview at any time without having to give a reason. Explain that they can choose whether to answer the questions or not now, and can opt out at any point during the conversation if they become uncomfortable.  Permission to record – for analysis purposes only  Gain informed consent on paper.  Ask participant to introduce her/himself:   - first name - age - how long they have lived in the community, and where is your family from? - Hobbies and what you like doing in your spare time? - Hopes for the future? | 10 mins |

| **YOUNG PEOPLE’S SEXUAL EXPERIENCES AND RELATIONSHIPS (theme 1)**  Why do young Aboriginal people have sex and/or get involved in sexual relationships?  *Feelings? Emotions? Urges?Enjoyment? Pleasure? Other benefits?*  Who do young Aboriginal people talk to about having sex? Why?  *Partner? Friends? Family? Elders? School or health services? Other?*  What kind of influences do Aboriginal family and community members have on Aboriginal young people’s sexual experiences or relationships? Why?  *Positive or negative? Support or discourage? Different for boys or girls? Or different ages?*  Can you give me some examples of the kinds of problems that Aboriginal young people might have resulting from having sex?  *Worries, concerns? Unexpected outcomes? Health problems? Trouble with friends or family or community?*    Can you tell me a story from your community about anything that we have talked about today? | 15 mins |
| --- | --- |
| **STIs AND RISK PRACTICES (theme 2)**  What can you tell me about sexually transmitted infections or disease amongst young Aboriginal people in your community?  *What are they? Any different examples? Signs and symptoms? Symptomatic vs asymptomatic? Good or bad? Why do young people get STIs? How cured?*  Which Aboriginal young people are more likely to get an STI? Why?  *Explore differences by sex, age, locality, sexuality*  What kind of things make some Aboriginal young people more likely to get an STI than others? Why?  *Use condoms? Lack of control in sex? Particular sexual practices? Lack of information? Lack of support from friends? Family? Health clinics? Sex whilst drunk/high?*  What happens when an Aboriginal young person gets an STI?  *Health consequences? Social consequences? Speak to anyone about it? Treated differently by others? Go to clinic? Why? Why not?*  Can you tell me a story from your community about anything that we have talked about today? | 15 mins |
| **PREVENTION, RISK REDUCTION AND STI TESTING/TREATMENT (theme 3)**  What are the main worries young Aboriginal people have about having sex or being involved in sexual relationships? Why?  *pregnancy? STIs? Gossip? Other?*  What do young Aboriginal people do to prevent sexual health problems?  *Increase awareness? Condom? Abstain? Fewer partners? Partner information? Visit sexual health centre? STI testing? Any other strategies?*  To what extent do young Aboriginal people use local sexual health services for STI testing or treatment? Why (not)?  *Socioecological Barriers? Symptomatic vs asymptomatic? Lack of knowledge? Bad previous experiences? Problems with health worker? Social stigma? Fear of being seen?*  What do you think could be done to increase STI testing and treatment among young Aboriginal people in this community??  *Clinic setting? Community setting? Awareness and education? Other support?*  Can you tell me a story from your community about anything that we have talked about today? | 15 mins |
| **Conclusions**  Explain that the interview is pretty much finished.  Are there any issues we haven’t discussed that you’d like to mention?  Explain that we would very much like to stay in touch about the progress of the project, and gather their insight along the way. As if that is okay? Get email and mobile number if needed.  Thank the interviewee for taking part.  Close interview, and stop recording. | 5 mins |
